# Unveiling the Origin of Alkali Metal (Na, K, Rb, and Cs) Promotion in CO_2_ Dissociation over Mo_2_C Catalysts

**DOI:** 10.3390/ma15113775

**Published:** 2022-05-25

**Authors:** Renmin Liu, Congmei Chen, Wei Chu, Wenjing Sun

**Affiliations:** 1 School of Chemical Engineering, Sichuan University, Chengdu 610065, China; 17806241710@163.com; 2 China-America Cancer Research Institute, Guangdong Provincial Key Laboratory of Medical Molecular Diagnostics, Guangdong Medical University, Dongguan 523808, China; 3 National Supercomputing Center in Shenzhen (Shenzhen Cloud Computing Center), Shenzhen 518055, China; chencm@nsccsz.gov.cn

**Keywords:** molybdenum carbide, alkali metal, RWGS, DFT

## Abstract

Molybdenum carbide (Mo_2_C) is a promising and low-cost catalyst for the reverse water−gas shift (RWGS) reaction. Doping the Mo_2_C surface with alkali metals can improve the activity of CO_2_ conversion, but the effect of these metals on CO_2_ conversion to CO remains poorly understood. In this study, the energies of CO_2_ dissociation and CO desorption on the Mo_2_C surface in the presence of different alkali metals (Na, K, Rb, and Cs) are calculated using density functional theory (DFT). Alkali metal doping results in increasing electron density on the Mo atoms and promotes the adsorption and activation of CO_2_ on Mo_2_C; the dissociation barrier of CO_2_ is decreased from 12.51 on Mo_2_C surfaces to 9.51–11.21 Kcal/mol on alkali metal-modified Mo_2_C surfaces. Energetic and electronic analyses reveal that although the alkali metals directly bond with oxygen atoms of the oxides, the reduction in the energy of CO_2_ dissociation can be attributed to the increased interaction between CO/O fragments and Mo in the transition states. The abilities of four alkali metals (Na, K, Rb, and Cs) to promote CO_2_ dissociation increase in the order Na (11.21 Kcal/mol) < Rb (10.54 Kcal/mol) < Cs (10.41 Kcal/mol) < K (9.51 Kcal/mol). Through electronic analysis, it is found that the increased electron density on the Mo atoms is a result of the alkali metal, and a greater negative charge on Mo results in a lower energy barrier for CO_2_ dissociation.

## 1. Introduction

Increasing atmospheric CO_2_ concentrations have resulted in global warming [1,2,3]. Therefore, CO_2_ capture, storage, and catalytic reduction have drawn attention to reduce this environmental burden [4]. In particular, the reverse water–gas shift (RWGS) reaction, which reduces CO_2_ to CO as an intermediate to generate methanol or other hydrocarbons, is promising [3]. The RWGS reaction is endothermic; thus, the RWGS reaction is thermodynamically favorable at high temperatures, as shown in Equation (1) [5].

(1) $$CO_{2}+H_{2}\to CO+H_{2}O\;\;\;\;\;\Delta H_{298K}^{{}^{\circ}}=41\;\mathrm{KJ}/\mathrm{mol}$$

Noble metal catalysts such as Pt [6,7], Rh [8,9], and Au [10] show reasonable activity and selectivity for the RWGS reaction but are costly. However, supported noble metal catalysts frequently suffer the problem of sintering under high temperature conditions. Moreover, noble metal catalysts are relatively expensive and scarce, which limits their ability to be widely used for CO_2_ hydrogenation. Transition metal carbides (TMCs) such as Mo_2_C [11,12], WC [13], and TiC [14] are considered as attractive candidates for the RWGS reaction because of their low cost and similar catalytic activity to platinum-based catalysts. TMCs have good performance in the reaction of CO_2_ conversion into CO [15], CH_4_ [16], CH_3_OH [17], and other hydrocarbons [18,19]. Among the carbide family members, molybdenum carbide (Mo_2_C) shows particularly high RWGS activity because of its favorable activity for C=O bond scission and H_2_ dissociation [20].

Alkali metals are very good promoters for CO_2_ conversion [21,22,23]. For example, CO_2_ activation was accelerated in K-modified CuxO/Cu (111) catalysts due to the geometric and electronic effects introduced by K [24]. The modification of Rh/Al_2_O_3_ with K changes the surroundings of the Rh particles, which influences the strength of CO adsorption and the activation ability of Rh for H_2_ dissociation [9]. The modification of Mo_2_C with alkali metals changes the structural and electronic properties of these catalysts and promotes the performances of Mo_2_C in CO_2_ conversions [25,26,27,28,29]. For example, the addition of 2 wt% K to Mo_2_C/γ-Al_2_O_3_ increases the CO selectivity to 95% from 73.5% [30], and the incorporation of K into Cu/Mo_2_C results in high CO_2_ dissociation activity (almost 1.5 times higher than Cu/Mo_2_C) but also reduces H_2_ adsorption, thus resulting in a low H_2_/CO*_x_* ratio and low CH_4_ production [31]. The CO selectivity of Cs-Mo_2_C, which can reach 100% at low-temperatures (400 to 500 °C), is a result of increased electron transfer from Cs to Mo, thus favoring CO selectivity [28]. By the introduction of K into the single atom catalyst Rh0.2/β-Mo_2_C, the selectivity of hydrogenation of CO_2_ to ethanol is much-improved, and the catalysts exhibit up to 72.1% of ethanol selectivity at low temperature (150 °C) [32].

Although the promotion effects of alkali metals on TMCs have been observed experimentally, the structural and electronic effects of alkali metals on TMCs in the RWGS reaction remain unknown. Additionally, different alkali metals affect the WGS and RWGS reactions to various extents. For instance, when Na and K species are introduced into WC, they both promote the improvement of the selectivity of WC for the RWGS reaction at low temperatures (300–350 °C), and the highest CO yield is achieved using K-promoted WC [13]. Kowalik et al. [33] reported that the promotional effect of alkali metals on the WGS activity of Cu/ZnO/Al_2_O_3_ catalysts increases in the order of Li < Na < K < Cs, but the promotional effects of H_2_O and CO_2_ dissociation induced by alkali metals increase in the order of Na < K < Rb < Cs over Cu (111) catalysts [22]. However, the reasons underlying these observations remain unknown.

In this study, we investigated the effect of the modification of the surface of Mo_2_C catalysts with alkali metals (Na, K, Rb, and Cs) using density functional theory calculations and revealed the key electronic effects affecting the adsorption of the reactants and intermediate species, the desorption of the products, as well as the barrier of CO_2_ dissociation. Furthermore, the energy barrier of CO_2_ dissociation was correlated with the adsorption energy of surface species, thus revealing the origin of the promoting effects of various alkali metals on RWGS activity. Our findings further highlight the importance of modifying molybdenum carbide with alkali for carbon dioxide reduction.

## 2. Computation Detail and Models

All calculations were performed by using the DMol^3^ code within the Materials Studio 7.0 program [34]. The generalized gradient approximation (GGA) with the Perdew–Burke–Ernzerhof (PBE) functional [35] was selected to calculate the exchange-correlation energy. The wave functions were expanded by the utilization of the double numerical quality basis set with polarization functions (DNP) [36]. The energy, gradient, and displacement convergence criteria were 1 × 10^−5^ hartree, 2 × 10^−3^ hartree/Å, and 5 × 10^−3^ Å, respectively.

LST/QST was used to perform the transition states (TS) search [37,38]. The convergence criterion of the TS search was set to 0.002 Ha/Å on each atom. Only one virtual frequency could be considered as the real transition state.

The adsorption energy ($E_{\mathrm{ads}}$) of all intermediate species on the surface of catalyst was defined as:

(2) $$E_{\mathrm{ads}}{=E}_{\mathrm{tot}}\;{-\;E}_{\mathrm{cat}}{\;-\;E}_{\mathrm{gas}}$$

where $E_{\mathrm{tot}}$ is the total energy of the adsorbed species on the catalyst, $E_{\mathrm{cat}}$ is the total energy of the clean catalyst, and $E_{\mathrm{gas}}$ is the energy of the molecules in the gas phase. The activation barrier ($E_{\mathrm{Barrier}}$) and reaction energy (∆E) were calculated using the formulas:

(3) $$E_{\mathrm{Barrier}}\;=E_{\mathrm{TS}}{\;-\;E}_{\mathrm{IS}}$$

(4) $$\text{∆}E=E_{\mathrm{FS}}{\;-\;E}_{\mathrm{IS}}$$

Here, $E_{\mathrm{IS}}$, $E_{\mathrm{TS}}$, and $E_{\mathrm{FS}}$ represent the total energies of the initial state (IS), transition state (TS), and the final state (FS), respectively. 

By applying geometry optimizations based on the minimization of the total energy of the unit cell, the DFT lattice parameters were found to be a = 6.00 Å, b = 5.78 Å, and c = 4.71 Å, which were in good agreement with the experimental results [39]. The slab model of the β-Mo_2_C (001) surface contained six atomic layers with a total of 24 C atoms and 48 Mo atoms in one unit cell (using a 2 × 2 supercell with size 12.00 × 11.57 × 4.71 Å, with a vacuum space of 20 Å). During the structural optimization, the bottom two layers were constrained in their bulk positions, whereas all the other atoms were allowed to relax. For alkali metal-modified β-Mo_2_C (001), one alkali atom (Na, K, Rb, Cs) was placed at different sites of the top layer of the molybdenum layer. After geometry optimization, the site that exhibited the strongest binding to K atom was selected for further calculations.

## 3. Results and Discussion

### 3.1. Optimized Structure of Alkali-Metal-Modified β-Mo_2_C (001)

**Figure 1:**
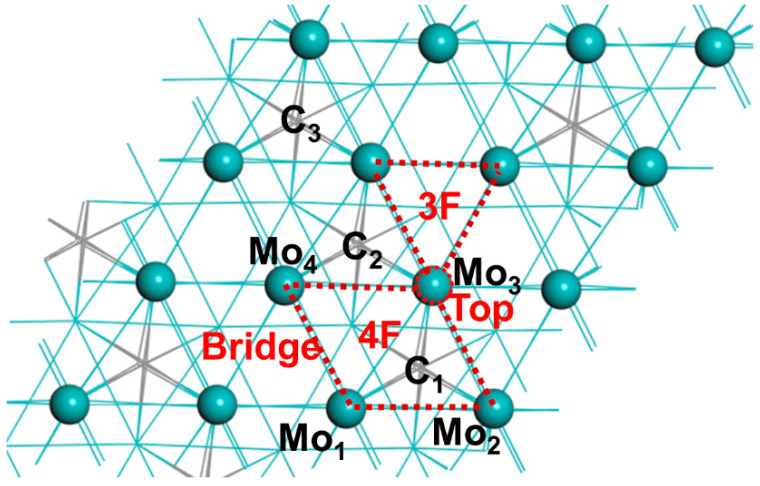
Position of the selected adsorption sites for alkali metal atoms. Color codes: Mo—green, carbon—grey, the first surface layer is shown using the ball and stick model, and the last five layers are shown using the line model.

**Figure 2:**
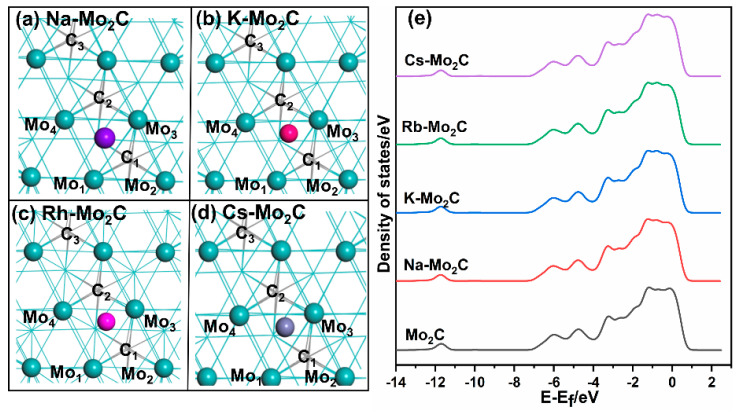
The most stable structure of X−Mo_2_C (001): (**a**) Na−Mo_2_C, (**b**) K−Mo_2_C, (**c**) Rb−Mo_2_C, and (**d**) Cs−Mo_2_C; (**e**) d−band center of Mo atoms on Mo_2_C and X-Mo_2_C (X = Na, K, Rb, and Cs). Color codes: Mo—green, carbon—grey (shown in line model).

The four alkali metal atoms at optimized structures of the X-Mo_2_C (X = Na, K, Rb, Cs) surfaces were all located on the 4F sites on the Mo_2_C surface, that is, between four Mo atoms (Figure 1). The alkali metal-promoted surfaces are shown in Figure 2a–d, and the key structural parameters of the X-Mo_2_C are listed in Table S1. The distances between alkali metals and Mo atoms increased with the increases in the atomic radii of the alkali metals: 3.26, 3.72, 3.89, and 4.04 Å on average for Na, K, Rb, and Cs, respectively. In addition, after the addition of the alkali metal, the Mo_1_–Mo_3_ and Mo_3_–M_4_ bonds increased in length by 0.04–0.06 Å, whereas the Mo_1_–Mo_4_ bond length was shortened by 0.03 Å for all X-Mo_2_C. The coverage of alkali metals on X-Mo_2_C surfaces was a 0.014 mono layer.

As shown by the charge analysis in Table 1, the Mulliken charge on Mo atoms in bare Mo_2_C was positive. In contrast, after the addition of the alkali metal, the charge on the adjacent Mo atoms became negative, suggesting the transfer of electrons from the alkali metal to Mo. Moreover, the closest Mo atoms to the alkali metal gained the most electrons. Because Rb and Cs atoms are less electronegative than the other alkali metals, they increased the electron density of the surface Mo atoms and subsurface C atoms. Therefore, in Rb- and Cs-Mo_2_C, the charges on Mo atoms were less negative than those of K and Na-Mo_2_C.

In addition, we calculated the changes in the d-band center [40] as a result of charge transfer between Mo and alkali metal atoms (Figure 2e). The d-band center before and after the addition of the alkali metal remained the same, consistent with previous findings [41,42].

**Table 1:** Mulliken charges (*e*) of key atoms in the clean and X-Mo_2_C (X = Na, K, Rb, Cs) *.

Catalysts	Mulliken Charge (*e*)						
	X	Mo_1_	Mo_3_	Mo_4_	C_1_	C_2_	C_3_
Mo_2_C	/	0.05	0.05	0.13	−0.50	−0.50	−0.50
Na-Mo_2_C	0.55	−0.08	−0.09	0.02	−0.47	−0.46	−0.45
K-Mo_2_C	0.72	−0.10	−0.09	0.03	−0.52	−0.51	−0.49
Rb-Mo_2_C	0.65	−0.06	−0.05	0.10	−0.61	−0.60	−0.60
Cs-Mo_2_C	0.66	−0.07	−0.06	0.09	−0.54	−0.53	−0.53

* Atom labels are indicated in Figure 1.

### 3.2. Adsorption of Intermediate Species on Mo_2_C and X-Mo_2_C Surfaces

**Figure 3:**
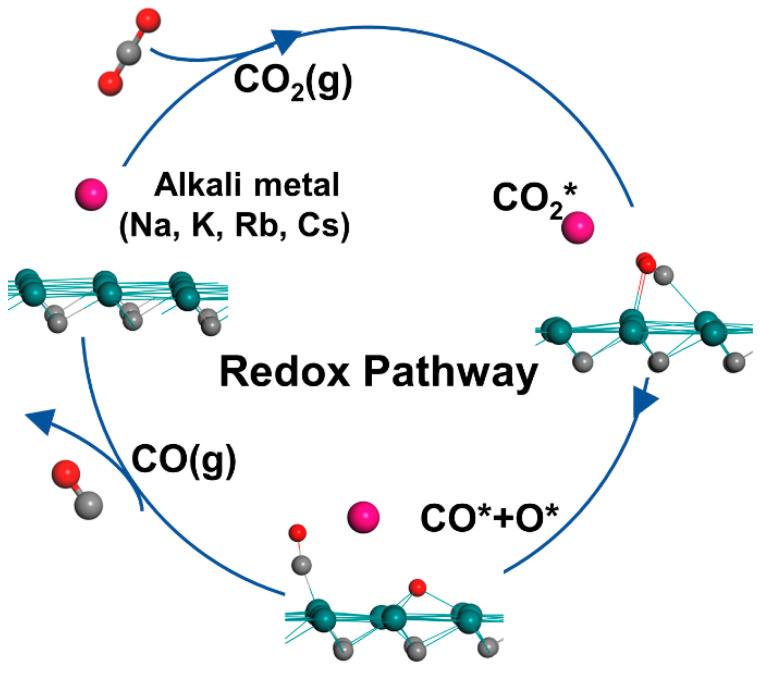
Schematic mechanism diagram of redox pathway in the RWGS reaction. The species with asterisks (*) represent adsorbed species.

**Figure 4:**
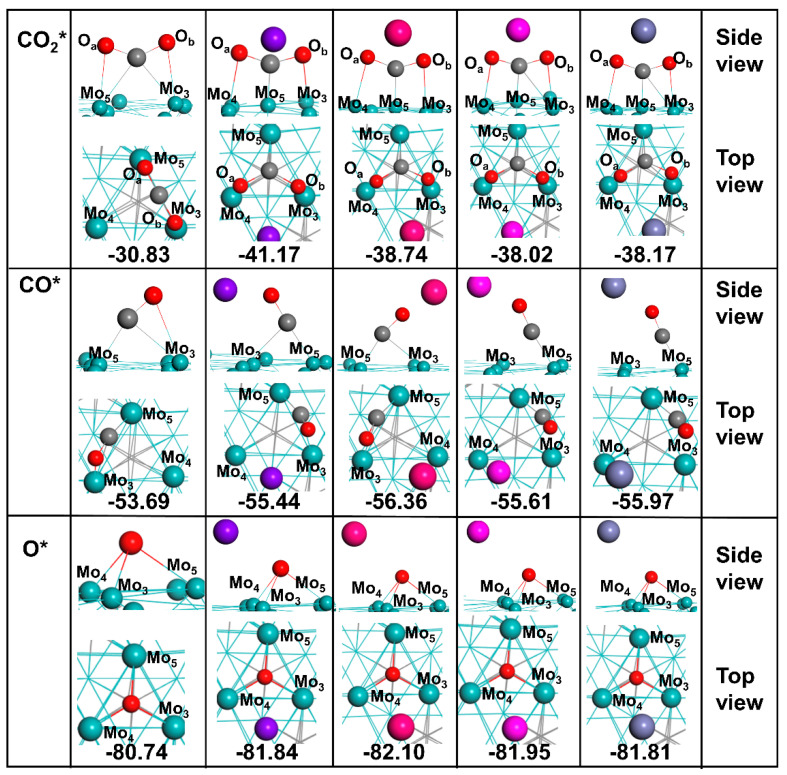
The most stable adsorption configurations of all possible surface intermediates as well as the adsorption energies (in Kcal/mol) on Mo_2_C and X−Mo_2_C (X = Na, K, Rb, and Cs). The species with asterisks (*) represent adsorbed species. Color codes: Mo—green, carbon—grey, O—red.

The schematic mechanism of the redox pathway is shown in Figure 3. We calculated the adsorption energy (*E*_ads_) and key parameters of CO_2_, CO, and O* species on bare and X-Mo_2_C surfaces involved in the redox pathway (shown in Figure 4 and Table S2).

#### 3.2.1. Adsorption of CO_2_*

The most stable adsorption configuration of CO_2_ on Mo_2_C is shown in Figure 4, having an adsorption energy of −30.83 Kcal/mol, consistent with the literature value [30]. The C atom in CO_2_ was positioned at the bridge site between Mo_3_ and Mo_5_, and the two C–Mo bonds were 2.24 Å in length. The nearest distance between the O and Mo atoms was 2.12 Å for O_a_–Mo_3_ and 2.43 Å for O_b_–Mo_5_. The interaction of CO_2_ with Mo_2_C led to the elongation of the C–O bonds from 1.17 Å in the vacuum calculations to 1.26 Å in the adsorbed molecule, and the bond angle increased to 135°.

For the adsorption of CO_2_ on all X-Mo_2_C surfaces, the carbon atom of CO_2_ was attached to the top site of one Mo atom, and the oxygen atoms formed two Mo–O bonds with the adjacent Mo atoms. The *E*_ads_ of CO_2_ was −41.17 Kcal/mol on Na-Mo_2_C, −38.74 Kcal/mol on K-Mo_2_C, −38.02 Kcal/mol on Rb-Mo_2_C, and −38.17 Kcal/mol on Cs-Mo_2_C. For CO_2_ adsorption on Na-Mo_2_C, the two O atoms in CO_2_ were equidistant from the Na atom (2.41 Å), CO_2_ was bent to 114°, and the C–O bonds were elongated to 2.41 Å. For the K-Mo_2_C catalyst, the K…O_a_ and K…O_b_ distances were found to be 2.74 and 2.97 Å, respectively, the CO_2_ bond was 115.6°, and the O–C bonds were elongated to 1.35 and 1.34 Å. For Rb-Mo_2_C, the Rb…O distances were 3.08 and 2.96 Å, and the C–O_a_ and C–O_b_ bond lengths were stretched to 1.34 and 1.36 Å, respectively. For Cs-Mo_2_, the Cs…O_a_ and Cs… O_b_ distances were 3.06 and 3.16 Å, respectively. Moreover, the C–O_a_ and C–O_b_ bond lengths were 1.33 and 1.36 Å, respectively. 

Thus, the X–O distances were close to the sum of the X^+^ and O^2−^ atomic radii, namely 2.41 Å for Na ^+^ +O^2−^, 2.77 Å for K^+^ + O^2−^, 2.91 Å for Rb^+^ + O^2−^, and 3.06 for Cs^+^ + O^2−^, as also observed for the X–O distances in crystalline metal oxides, for example, 2.40 Å in Na_2_O [43], 2.79 in K_2_O [43], 2.92 in Rb_2_O [43], and 3.26 Å in CsO_2_ [44]. These findings indicate that the electrostatic interaction between O and X was strong and similar to the ionic bonding between O and X in X_2_O. Wang et al. found that for oxygenate species adsorbed on K^+^-modified Cu (111) and Cu (110) surfaces, when the distance between K and O atoms was 3.00 Å, direct bonding between O^δ−^ and K^δ+^ ions occurred [22]. Further, a short distance between X and O generated a longer C–O bond, indicating that stronger X–O interactions promote CO_2_ activation. Therefore, alkali metals could promote the adsorption and activation of CO_2_, consistent with theoretical and experimental findings [30].

Our charge analysis (Table S3) suggested that electrons are transferred from the alkali metal to Mo atoms and, thus, affect the surface charge of Mo_2_C [28]. Therefore, when CO_2_ was adsorbed on Mo_2_C, the Mo atoms lost electrons and CO_2_ gained 0.29 *e*. In contrast, when CO_2_ was adsorbed on X-Mo_2_C, the Mo atoms became more positive (lost more electrons); for example, CO_2_ gained 0.74 *e* on Na-Mo_2_C, 0.71 *e* on K-Mo_2_C, 0.73 *e* on Rb-Mo_2_C, and 0.69 *e* on Cs-Mo_2_C. The increase in the charge of CO_2_ also indicated enhanced charge transfer via alkali metal promotion. Moreover, in the X-Mo_2_C surfaces, alkali metal atoms lost electrons by 0.72 *e* for Na, 0.82 *e* for K, 0.76 *e* for Rb, and 0.74 *e* for Cs, respectively. Therefore, on one hand, alkali metal atoms donated electrons to CO_2_, but, on the other hand, they facilitated electron transfer from Mo to CO_2_ and thus promoted CO_2_ activation.

#### 3.2.2. Adsorption of CO*

When CO adsorbed on the Mo_2_C surface, the carbon atom of CO_2_ was adsorbed on the bridge sites between Mo_3_ and Mo_5_ with an orientation tilted toward Mo_4_. The generated C–Mo_4_, C–Mo_5_, and O–Mo_4_ bond lengths were 2.27, 1.99, and 2.35 Å, respectively. Further, the C–O bond length was elongated to 1.23 Å, and the *E*_ads_ of CO on Mo_2_C was −53.69 Kcal/mol.

The *E*_ads_ values of CO on X-Mo_2_C (X = Na, K, Rb, and Cs) were around −56 Kcal/mol, approximately 1.75–2.67 Kcal/mol greater than that on Mo_2_C. The maximum differences in *E*_ads_ on the X-Mo_2_C surfaces were within 1 Kcal/mol, i.e., negligible. In these systems, CO was adsorbed in a tilted orientation on the bridge sites of Mo_3_–Mo_5_ atoms with the oxygen atom oriented towards the Mo_3_ atom. The distances between alkali metal and O (in CO) were 2.42 Å for Na…O, 2.78Å for K…O, 2.93 Å for Rb…O, and 3.18 Å for Cs…O, suggesting that the alkali metals formed direct bonds with the O atom in CO in X-Mo_2_C systems. The Mulliken charge analysis (Table S4) showed that more electrons transferred to CO on X-Mo_2_C than that on bare Mo_2_C. Therefore, the addition of alkali metal atoms increased the adsorption of CO as compared with the clean surface and resulted in longer C–O bonds.

#### 3.2.3. Adsorption of O*

Atomic oxygen adsorbed on the 3F sites between the Mo_3_–Mo_4_–Mo_5_ atoms, having average Mo–O bond lengths of 2.08 Å and *E*_ads_ of −80.74 Kcal/mol. The *E*_ads_ for O* on the surface of X-Mo_2_C (X = Na, K, Rb, and Cs) ranged from −81.64 to −82.10 Kcal/mol, slightly greater than that on bare Mo_2_C. The average distances between O* and Mo_3_ atoms were 2.46 Å for Na, 2.85Å for K, 3.03 Å for Rb, and 3.04 Å for Cs. On bare Mo_2_C, the atomic oxygen gained 0.66 *e* from the surface of Mo atoms, whereas for X-Mo_2_C, the O atom gained more electrons, namely 0.74 *e* from Na-Mo_2_C, 0.73 *e* from K-Mo_2_C, 0.72 *e* from Rb-Mo_2_C, and 0.73 *e* from Cs-Mo_2_C (see Table S5). Moreover, the Mo atoms in the X-Mo_2_C surfaces were more positive, suggesting that the addition of alkali metals promoted the loss of electrons from the Mo atoms around O*. 

### 3.3. Energy Barriers for CO_2_ Dissociation on Mo_2_C and Alkali-Metal-Modified Mo_2_C Surfaces

Currently, the RWGS reaction mechanisms are classified into redox- (or direct-), carboxyl-, and formate-mediated routes. Chen et al. [45] performed ambient-pressure X-ray photoelectron spectroscopy (AP-XPS) measurements on the Mo_2_C catalyst, and they did not find intermediate species (carbonate, formate, carbonyl, etc.) under reaction conditions. Furthermore, they proved that CO_2_ was directly dissociated on Mo_2_C to produce CO and oxycarbide (Mo_2_C-O). Surface oxygen (Mo_2_C-O) was removed subsequently by hydrogen to produce H_2_O to complete the catalytic cycle [46]. Moreover, the elemental steps for CO_2_ dissociation to CO* and O* are known to be the rate limiting steps on both bare Mo_2_C and K-modified Mo_2_C catalysts [30]. Thus, the activation barriers for CO_2_ dissociation on the various alkali metal-modified Mo_2_C surfaces were studied and compared. 

**Figure 5:**
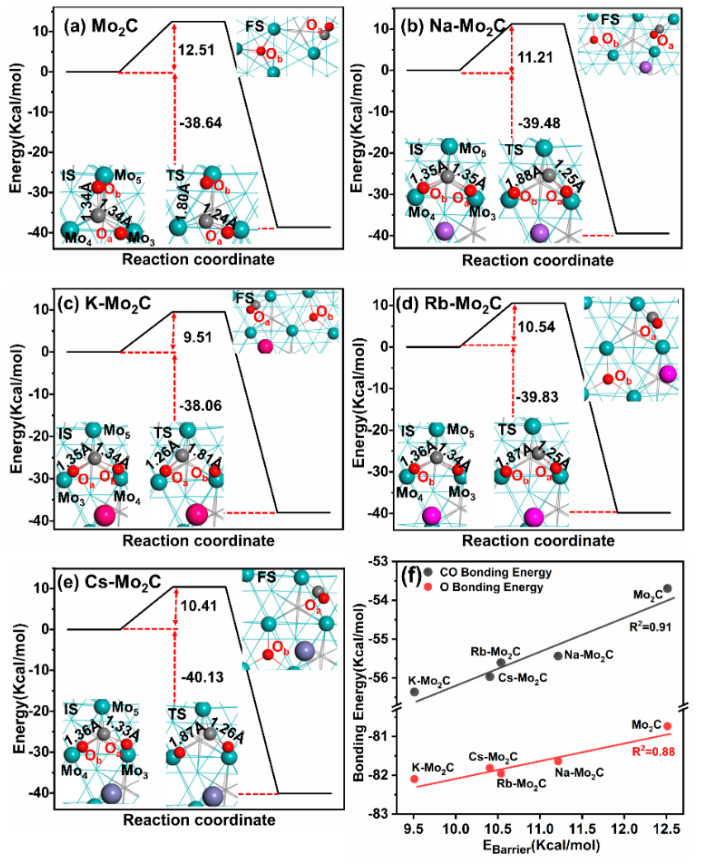
CO_2_ dissociation profiles on (**a**) Mo_2_C, (**b**) Na−Mo_2_C, (**c**) K−Mo_2_C, (**d**) Rb−Mo_2_C, and (**e**) Cs−Mo_2_C. (**f**) Correlation between activation energy of the CO_2_ dissociation and CO/O bonding energy.

In Figure 5, we show the activation energy profiles of CO_2_ dissociation on the (a) bare, (b) Na-promoted, (c) K-promoted, (d) Rb-promoted, and (e) Cs-promoted Mo_2_C (001) surfaces. The activation barrier for CO_2_ dissociation on Mo_2_C surfaces was found to be 12.51 Kcal/mol, and the O–CO bond lengths of the transition states (TSs) were found to be 1.75 Å, indicating the cleavage of a C–O bond. The activation energies for this reaction were remarkably different on the four X-Mo_2_C surfaces, namely 11.21 Kcal/mol for Na-Mo_2_C, 9.51 Kcal/mol for K-Mo_2_C, 10.54 Kcal/mol for Rb-Mo_2_C, and 10.41 Kcal/mol for Cs-Mo_2_C, lower than that on bare Mo_2_C. For CO_2_ dissociation on the X-Mo_2_C surfaces, the bond length of C–O_b_ was elongated, ranging from 1.81 to 1.87 Å in the TS, respectively, longer than that on bare Mo_2_C. In addition, the distance between Na, K, Rb, and Cs and O_a_ in CO_2_ were 2.14, 2.94, 3.07, and 3.06 Å, respectively, suggesting the interaction between X and O throughout the reaction and suggesting the key role of the alkali metal in CO_2_ dissociation, consistent with experimental observations [47,48,49].

Figure 5 suggests that the transition state for CO_2_ dissociation is a late (product-like) transition state. Therefore, for CO_2_ dissociation, the stabilization of the final state should also stabilize the transition state, resulting in a lower activation barrier. Figure 5f shows that the CO_2_ dissociation barrier was strongly influenced by the $E_{\mathrm{ads}}$ of CO and O fragments on the catalyst surfaces, and stronger CO or O binding resulted in lower CO_2_ dissociation barriers. Therefore, the identification of the role of the alkali metal atom on the stability of adsorbed CO and O during the reaction is necessary.

### 3.4. Energetic Analysis

As discussed earlier, alkali metals enhance the RWGS activity of Mo_2_C by decreasing the energy barriers for CO_2_ dissociation. Thus, to elucidate the effects of the alkali metals, the physical origin of the reaction barriers for CO_2_ dissociation on both bare and X-Mo_2_C surfaces were assessed using energy decomposition, as proposed by Hammer [50,51] (Equation (4)), and the results are listed in Table 2.

(5) $$E_{\mathrm{Barrier}}{=E}_{\mathrm{bond}}^{{\mathrm{CO}}_{2}}{\;-\;E}_{{\mathrm{CO}}_{2}}^{\mathrm{IS}}{+E}_{\mathrm{CO}}^{\mathrm{TS}}{+E}_{O}^{\mathrm{TS}}{+E}_{\mathrm{int}}^{\mathrm{TS}}\;$$

where $E_{\mathrm{bond}}^{{\mathrm{CO}}_{2}}$ represents the bonding energy of CO_2_ in gas. $E_{{\mathrm{CO}}_{2}}^{\mathrm{IS}}$, $E_{\mathrm{CO}}^{\mathrm{TS}}$, $E_{O}^{\mathrm{TS}}$, and $E_{\mathrm{int}}^{\mathrm{TS}}\;$ refer to binding energy of CO_2_* in the IS, binding energy of CO* (O*) in the TS, and the interaction of CO with O in the TS, respectively.

**Figure 6:**
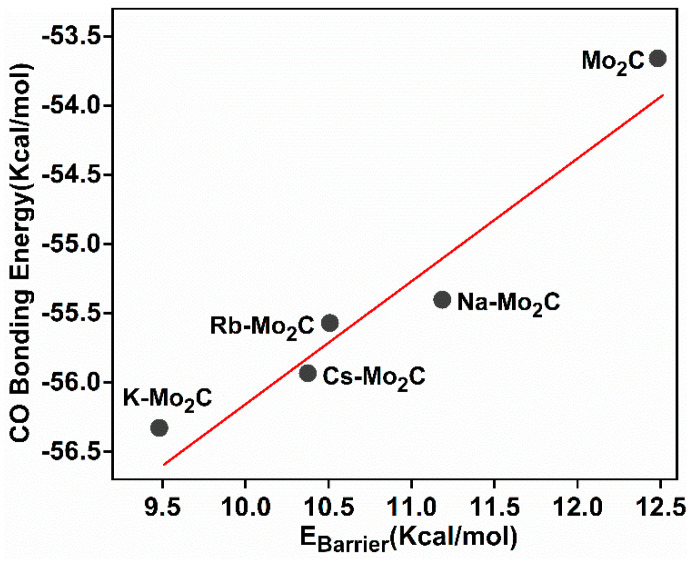
Correlation between activation energy of the CO_2_ dissociation and MaxΔ$E_{S}^{\mathrm{TS}}$ (fitting model: y = 2.16x − 26.56; R^2^ = 0.90).

In the C–O bond scission of CO_2_ on Mo_2_C surfaces, alkali metals stabilize the binding of CO_2_ in the IS, which is unfavorable for reducing the energy barrier (Table 2). However, by strengthening CO and O binding in the TS ($E_{\mathrm{CO}}^{\mathrm{TS}}\;$ and $E_{O}^{\mathrm{TS}}$) on X-Mo_2_C relative to those on clean surfaces, the alkali metal reduces the energy barrier and promote CO_2_ dissociation. In addition, all alkali metals can enhance the stability of CO or O fragments in the TS on Mo_2_C. However, Na- and K-modified surfaces effectively stabilize adsorbed CO compared to bare Mo_2_C (${\Delta E}_{\mathrm{CO}}^{\mathrm{TS}}>{\Delta E}_{O}^{\mathrm{TS}})$, whereas Rb- and Cs-modified surfaces stabilize adsorbed O (${\Delta E}_{O}^{\mathrm{TS}}>{\Delta E}_{\mathrm{CO}}^{\mathrm{TS}})$. Therefore, Max${\Delta E}_{S}^{\mathrm{TS}}$, i.e., the maximum of ${\Delta E}_{\mathrm{CO}}^{\mathrm{TS}}$ and ${\Delta E}_{O}^{\mathrm{TS}}$ was plotted against the energy barrier for CO_2_ dissociation. Figure 6 shows that the energy barrier for CO_2_ dissociation on X-Mo_2_C was linearly correlated to Max${\Delta E}_{S}^{\mathrm{TS}}$ (*R*^2^ = 0.90), and a greater value of Max${\Delta E}_{S}^{\mathrm{TS}}$ indicated a larger decrease of the barrier and a stronger promoting effect of the alkali metal. In other words, increasing the $E_{\mathrm{ads}}$ of the CO and O fragments in the TS can effectively reduce the energy barrier, but the extent of the barrier reduction depends on the balance of stabilities of adsorbed CO and O resulting from alkali metal addition.

As displayed in Figure 5, the alkali metals interacted with CO and O on the X-Mo_2_C surfaces throughout the reaction. Hence, apart from the interaction between these adsorbates and surface Mo atoms, the interaction between them and the alkali metal adatom also made up the interaction of them with X-Mo_2_C.The interaction energies of the interactions between adsorbates and alkali metals were calculated with the following formulas, and the results are listed in Table 3.

(6) $$E_{\mathrm{int}}^{A-X}{=E}_{A/X}^{\mathrm{TS}}{\;-\;(E}_{A}{+\mathrm{Ex}\;-\;E}_{\mathrm{surf}}^{\mathrm{TS}})$$

(7) $$E_{\mathrm{int}}^{A-\mathrm{Mo}}{=E}_{A}^{\mathrm{TS}}{\;-\;E}_{\mathrm{int}}^{A-X}$$

$E_{A/X}^{\mathrm{TS}}$, $E_{A}$, and Ex represent the energies of the A−alkali metal complex, isolated A, and alkali metal at Mo_2_C surfaces, respectively; and $E_{\mathrm{surf}}^{\mathrm{TS}}$ refers to the energy of the clean surface at the TS. 

As shown in Table 3, the addition of alkali metal adatoms resulted in interactions between the alkali metal and oxygen species in the TS, but there was little correlation between $E_{\mathrm{int}}^{\mathrm{CO}-X}$/$E_{\mathrm{int}}^{O-X}$ and $E_{\mathrm{Barrier}}$. However, the addition of alkali metals increased the strength of the O−Mo or CO–Mo bonds, as shown by the greater $E_{\mathrm{int}}^{\mathrm{CO}-\mathrm{Mo}}$ and $E_{\mathrm{int}}^{O-\mathrm{Mo}}$ on X-Mo_2_C compared to those on bare Mo_2_C. In addition, the increase in the CO–Mo and O–Mo interactions in X-Mo_2_C ($E_{\mathrm{int}}^{\mathrm{CO}-\mathrm{Mo}}$ and $E_{\mathrm{int}}^{O-\mathrm{Mo}}$) was linearly related to the increase in the bonding energies of CO (${\Delta E}_{\mathrm{CO}}^{\mathrm{TS}})\;$ and O (${\Delta E}_{O}^{\mathrm{TS}})$ in the TS. Therefore, the increased bonding energies of CO and O in the TS were a result of the increased Mo–O and Mo–CO bond strength resulting from the addition of alkali metals.

Previous studies have demonstrated that the activation of CO_2_ requires electron transfer from the catalyst to CO_2_ [52,53,54,55]. The charge analysis in Table 3 shows that the enhancement in the Mo–O and Mo–CO interactions was due to the addition of alkali metals, which induced the accumulation of electron density at Mo and, thus, electron transfer from Mo to CO and O in the TS. Further, we observed a linear increase in the negative charge on the Mo atoms (increase in electron density) in the Na, Rb, Cs, and K-Mo_2_C surfaces, which was consistent with the reduction in $E_{\mathrm{Barrier}}$. This result indicated that the increase in charge at Mo resulted in a lower $E_{\mathrm{Barrier}}$. Additionally, although Rb and Cs are less electronegative than the other alkali metals, they transferred electrons to both Mo and C in the subsurface; thus, fewer electrons accumulated at Mo in Rb/Cs-Mo_2_C than in K-Mo_2_C. 

In summary, the CO_2_ dissociation energy barrier was in the order of Mo_2_C (12.45 Kcal/mol) > Na (11.21 Kcal/mol) > Rb (10.54 Kcal/mol) > Cs (10.41 Kcal/mol) > K(9.51 Kcal/mol). This is because the K atom promoted the most electrons accumulated at the Mo atom and thereby generated the strongest Mo–CO interactions in the TS. The significantly improved stability of CO fragments in the TS led to the energy barrier of CO_2_ dissociation on K-Mo_2_C being the lowest.

**Table 3:** Mulliken charges (*e*) of key atoms in the calculated transition states.

	Mo		CO_2_			
	Mo_1_ ^a^	Mo_2_ ^b^	O_a_	C	O_b_	Alkali Metal
Mo_2_C	0.19	0.30	−0.30	0.15	−0.50	
Na-Mo_2_C	0.12	0.17	−0.41	0.19	−0.61	0.74
K-Mo_2_C	0.06	0.23	−0.42	0.16	−0.60	0.83
Rb-Mo_2_C	0.20	0.19	−0.39	0.13	−0.58	0.77
Cs-Mo_2_C	0.18	0.18	−0.40	0.18	−0.59	0.76

^a^ Mo atom that bonded with O_a_, ^b^ Mo atom that bonded with O_b_.

### 3.5. CO Desorption on Mo_2_C and Alkali-Metal-Modified Mo_2_C Surfaces

**Figure 7:**
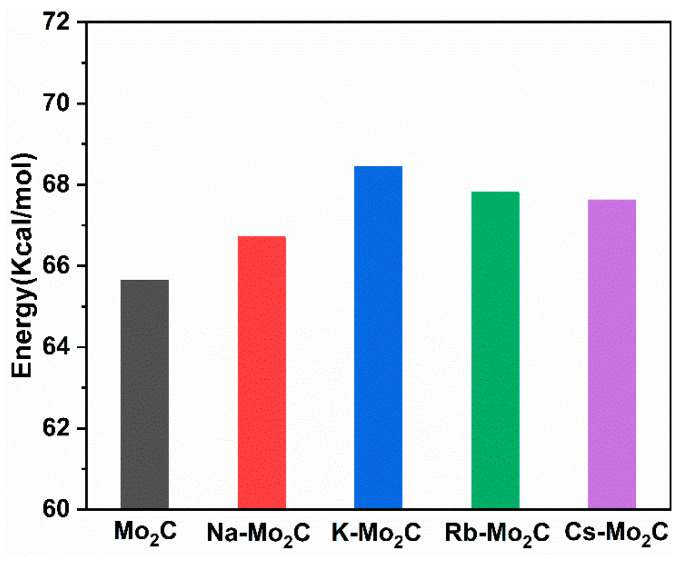
The desorption energy of CO on bare and X-Mo_2_C, where X = Na, K, Rb, Cs.

Next, we calculated the energies of CO desorption on the surfaces of bare Mo_2_C and X-Mo_2_C (Figure 7). The CO desorption energy on bare Mo_2_C was endothermic by 63.65 Kcal/mol. The addition of alkali metals on Mo_2_C slightly increased the difficulty of CO desorption. Mpourmpakis et al. found that by pre-adsorption of low-coverage oxygen (<0.50 ML), the desorption of CO on K-modified Mo_2_C could be effectively promoted [56].

## 4. Conclusions

The activity of CO_2_ dissociation into CO on bare Mo_2_C and those on promoted surfaces of X-Mo_2_C (X = Na, K, Rb, and Cs) were studied using DFT calculations. The addition of alkali metal elements induced the accumulation of negative charges on the Mo atoms and thus promoted the adsorption and activation of CO_2_ on Mo_2_C. The CO_2_ dissociation energy barrier was in the order of Mo_2_C (12.45 Kcal/mol) > Na (11.21 Kcal/mol) > Rb (10.54 Kcal/mol) > Cs (10.41 Kcal/mol) > K (9.51 Kcal/mol). On the basis of energetic and electronic analysis, although the alkali metals directly bonded with oxygen atoms in the adsorbed oxygen species, the main reason for the reduction in the energy of CO_2_ dissociation was the stronger interaction between CO/O fragments and Mo in the TS. Through electronic analysis, the promoting effects of alkali metals were influenced by the difference in the increase of electron density at the Mo atoms. Specifically, the greater the negative charge on the Mo site, the lower the energy barrier for CO_2_ dissociation. In comparison, the K atom promoted the most electrons accumulated at the Mo atom and thereby generated the strongest Mo–CO interactions in the TS. The significantly improved stability of CO fragments in the TS led to the energy barrier of CO_2_ dissociation on K-Mo_2_C being the lowest.

## Figures and Tables

**Table 2:** Energy decomposition of the calculated activation barriers for the dissociation of CO_2_ on bare Mo_2_C and X-Mo_2_C.

Catalysts	$E_{\mathrm{Barrier}}$	$E_{{\mathrm{CO}}_{2}}^{\mathrm{IS}}$	$E_{\mathrm{CO}}^{\mathrm{TS}}$	$E_{O}^{\mathrm{TS}}$	$E_{\mathrm{int}}^{\mathrm{TS}}$	$E_{\mathrm{int}}^{\mathrm{CO}-X}{\;}^{a}$	$E_{\mathrm{int}}^{\mathrm{CO}-\mathrm{Mo}}$	$E_{\mathrm{int}}^{O-X}{}^{\;a}$	$E_{\mathrm{int}}^{O-\mathrm{Mo}}$	$\Delta E_{CO}^{TS}{\;}^{b}$	$\Delta E_{O}^{TS}{\;}^{b}$
Mo_2_C	12.45	−50.78	−44.89	−72.33	−68.64	0.00	−44.89	0.00	−72.33	0.00	0.00
Na-Mo_2_C	11.30	−60.28	−47.39	−74.22	−75.04	−0.09	−47.29	−0.28	−73.94	−2.50	−1.89
K-Mo_2_C	9.45	−60.44	−51.82	−73.31	−73.38	−0.12	−51.71	−0.32	−73.00	−6.93	0.99
Rb-Mo_2_C	10.61	−61.34	−47.14	−75.08	−76.17	−0.12	−47.03	−0.25	−74.82	−2.25	−2.75
Cs-Mo_2_C	10.38	−60.11	−45.36	−76.04	−75.89	−5.77	−45.32	−0.25	−75.77	−0.47	−3.71

^a^ The interaction energies of A-X as well as between A and Mo atoms at the TSs (A refers to adsorption species). The C−O bonding energy of CO_2_ in the gas phase is calculated to be 147.59 Kcal/mol. ^b^ Δ$E_{A}^{\mathrm{TS}}=E_{A,\;X-{\mathrm{Mo}}_{2}C\;}^{\mathrm{TS}}$ − $E_{A,\;{\mathrm{Mo}}_{2}C\;}^{\mathrm{TS}}$.

## Data Availability

The data presented in this study are available from the corresponding author, upon reasonable request.

## Supplementary Materials

The following supporting information can be downloaded at: https://www.mdpi.com/article/10.3390/ma15113775/s1, Table S1: The key structural parameters of Mo_2_C and X-Mo_2_C; Table S2: Adsorption energies (Kcal/mol) and bond length (Å) of all possible intermediates on the X (Na, K, Rb, Cs)-Mo_2_C catalysts; Table S3: Mulliken charge; Table S4: Mulliken charge (*e*) of CO*-X-Mo_2_C; Table S5: Mulliken charge (*e*) of O*-X-Mo_2_C.
