# Supplementary material for: Unveiling the Origin of Alkali Metal (Na, K, Rb, and Cs) Promotion in CO2 Dissociation over Mo2C Catalysts
Source: Materials (Basel). 2022 May 25;15(11):3775. doi: 10.3390/ma15113775 (PMC9181518; doi:10.3390/ma15113775)
Supplement: Supplementary file 1 [file materials-15-03775-s001.zip › materials-1697774-supplementary.pdf]

Unveiling the origin of alkali metal(Na, K, Rb, and Cs) promotion in CO_2 dissociation over Mo_2C catalysts

Renmin Liu [1,2], Congmei Chen [3], Wei Chu [1,*] and Wenjing Sun [2,*]

[1] School of Chemical Engineering, Sichuan University, Chengdu 610065, Sichuan, China

[2] China-America Cancer Research Institute, Key Laboratory for Medical Molecular Diagnostics of Guangdong Province, Guangdong Medical University, Dongguan 523808,

[3] Guangdong, China National Supercomputing Center in Shenzhen (Shenzhen Cloud Computing Center), Shenzhen 518055, Guangdong, China

*Correspondence: E-mail: chuwei1965@foxmail.com(Wei Chu); swj_gdmc@163.com (Wenjing Sun)

Content

Table S1 The key structural parameters of Mo_2C and X-Mo_2C..…………………....3

Table S2 Adsorption energies and bond length of all possible intermediates on the X-Mo_2C catalysts……………………………4

Table S3 Mulliken charge(*e*) of CO_2*-X-Mo_2C…………………………...…….5

Table S4 Mulliken charge(*e*) of CO*-X-Mo_2C…………….……………...…….6

Table S5 Mulliken charge(*e*) of O*-X-Mo_2C…………………………..…...…….7

Table S1 The key structural parameters of Mo_2C and X-Mo_2C

Catalysts	Bond lengths/Å
Mo_2C	Mo_1-Mo_3 3.14, Mo_1-Mo_4 2.88, Mo_3-Mo_4 3.09
Na-Mo_2C	Na-Mo_1 3.27, Na-Mo_3 3.22, Na-Mo_4 3.30,
	Mo_1-Mo_3 3.18, Mo_1-Mo_4 2.85, Mo_3-Mo_4 3.14
K-Mo_2C	K-Mo_1 3.84, K-Mo_3 3.58, K-Mo_4 3.75,
	Mo_1-Mo_3 3.18, Mo_1-Mo_4 2.85, Mo_3-Mo_4 3.15
Rb-Mo_2C	Rb-Mo_1 4.02, Rb-Mo_3 3.74, Rb-Mo_4 3.94,
	Mo_1-Mo_3 3.18, Mo_1-Mo_4 2.85, Mo_3-Mo_4 3.15
Cs-Mo_2C	Cs-Mo_1 4.16, Cs-Mo_3 3.87, Cs-Mo_4 4.09,
	Mo_1-Mo_3 3.18, Mo_1-Mo_4 2.86, Mo_3-Mo_4 3.14

Table S2 Adsorption energies (Kcal/mol) and bond length (Å) of all possible intermediates on the X(Na,K,Rb,Cs)-Mo_2C catalysts

	Species	E_{ads}(Kcal/mol)	Bond length(Å)
Mo_2C	CO_2	-30.83	C-Mo_3 2.24, C-Mo_3 2.24,O_a- Mo_3 2.12, O_b-Mo_5 2.43, C-O_a 1.26, C-O_b 1.26,
	CO	-53.69	C-Mo_4 2.27, C-Mo_5 1.99, O-Mo_4 2.35, C-O 1.23
	O	-80.74	O-Mo3 2.10, O-Mo4 2.09, O-Mo5 2.04
Na-Mo_2C	CO_2	-41.17	C-Mo_5 2.17, O_a-Mo_3 2.13, O_b-Mo_4 2.12, C-Oa 1.35, C-Ob 1.35, Na-Oa 2.41, Na-Ob 2.41
	CO	-55.44	C-Mo_3 2.39, C-Mo_5 2.00, O-Mo_3 2.93, C-O 1.22, Na-O 2.42
	O	-81.84	O-Mo3 2.15, O-Mo4 2.15, O-Mo5 2.06, Na-O 2.39
K-Mo_2C	CO_2	-38.74	C-Mo_5 2.17, O_a-Mo_3 2.13, O_b-Mo_4 2.12, C-Oa 1.35, C-Ob 1.35, K-Oa 2.74, K-Ob 2.97
	CO	-56.36	C-Mo_4 2.26, C-Mo_5 1.98, O-Mo_4 2.40, C-O 1.25, K-O 2.78
	O	-82.10	O-Mo3 2.14, O-Mo4 2.13, O-Mo5 2.06, K-O 2.85
Rb-Mo_2C	CO_2	-38.02	C-Mo_5 2.19, O_a-Mo_3 2.13, O_b-Mo_4 2.09, C-Oa 1.34, C-Ob 1.36, Rb-Oa 3.08, Rb-Ob 2.96
	CO	-55.61	C-Mo_3 2.42, C-Mo_5 2.00, O-Mo_3 3.04, C-O 1.25, Rb-O 2.93
	O	-81.95	O-Mo3 2.13, O-Mo4 2.13, O-Mo5 2.06, Rb-O 3.03
Cs-Mo_2C	CO_2	-38.17	C-Mo_5 2.16, O_a-Mo_3 2.17, O_b-Mo_4 2.09, C-Oa 1.33, C-Ob 1.36, Cs-Oa 3.06, Cs-Ob 3.16
	CO	-55.97	C-Mo_3 2.42, C-Mo_5 2.01, O-Mo_3 3.04, C-O 1.21, Cs-O 3.18
	O	-81.81	O-Mo3 2.14, O-Mo4 2.13, O-Mo5 2.06, Cs-O 3.04

Table S3 Mulliken charge(e) of CO_2*-X-Mo_2C

Atom	Mo_2C	Na-Mo_2C	K-Mo_2C	Rb-Mo_2C	Cs-Mo_2C
Mo_1	0.08	-0.12	-0.11	-0.07	-0.08
Mo_2	0.16	0.11	0.11	0.17	0.14
Mo_3	0.10	0.17	0.17	0.22	0.20
Mo_4	0.10	0.09	0.08	0.11	0.09
Mo_5	0.10	0.10	0.11	0.14	0.12
Mo_6	0.13	0.09	0.12	0.15	0.15
O_a	-0.36	-0.52	-0.51	-0.51	-0.49
C	0.43	0.30	0.31	0.25	0.32
O_b	-0.36	-0.52	-0.51	-0.48	-0.52
Alkali metal	/	0.72	0.82	0.76	0.74

Table S4 Mulliken charge(*e*) of CO*-X-Mo_2C

Atom	Mo_2C	Na-Mo_2C	K-Mo_2C	Rb-Mo_2C	Cs-Mo_2C
Mo_1	0.04	-0.08	-0.09	-0.04	-0.04
Mo_2	0.14	0.09	0.10	0.13	0.12
Mo_3	0.18	0.08	0.07	0.20	0.18
Mo_4	0.09	-0.04	-0.02	-0.05	-0.05
Mo_5	0.08	0.03	0.07	0.10	0.06
Mo_6	0.20	0.10	0.16	0.14	0.12
O	-0.28	-0.33	-0.40	-0.31	-0.31
C	0.04	0.08	0.00	-0.02	0.02
Alkali metal	/	0.65	0.80	0.73	0.76

Table S5 Mulliken charge(e) of O*-X-Mo_2C

Atom	Mo_2C	Na-Mo_2C	K-Mo_2C	Rb-Mo_2C	Cs-Mo_2C
Mo_1	0.06	-0.09	-0.10	-0.07	-0.07
Mo_2	0.13	0.09	0.09	0.13	0.12
Mo_3	0.30	0.18	0.20	0.25	0.24
Mo_4	0.27	0.15	0.15	0.17	0.16
Mo_5	0.32	0.26	0.27	0.30	0.28
O	-0.66	-0.74	-0.73	-0.72	-0.73
Alkali metal	/	0.67	0.79	0.72	0.73
